# The Integration of Music Therapy in the Intensive Care Unit: Health Care Professionals' Experience of an Emerging Paradigm

**DOI:** 10.1002/nop2.70994

**Published:** 2026-11-03

**Authors:** Verónica Saldaña‐Ortiz, María Caballero‐Galilea, Vanesa Intriago‐García Consuegra, José Miguel Mansilla‐Domínguez, Beatriz García‐Fernández, Esther Martínez‐Miguel

**Affiliations:** ^1^ Department of Nursing, Faculty of Medicine, Health and Sports Universidad Europea de Madrid Madrid Spain; ^2^ Department of Nursing Universidad Antonio de Nebrija Facultad de Ciencias de la Vida y de Naturaleza Madrid Spain

**Keywords:** anxiety, critical care, health care professionals, music therapy

## Abstract

**Objective:**

To explore how intensive care unit healthcare professionals experienced and interpreted music therapy delivered to critically ill patients, including its perceived relevance to patient experience, care interactions, the ICU environment and their own professional experience during care.

**Design:**

A qualitative study with a hermeneutic phenomenological approach.

**Methods:**

A qualified music therapist delivered live, individualised music therapy sessions to critically ill patients alongside ongoing ICU care, including selected procedures when clinically appropriate. Fourteen healthcare professionals who had each been present during one patient‐directed session participated in an individual semi‐structured interview 24–48 h later. Data were analysed using reflexive thematic analysis.

**Results:**

Four themes were developed: (1) Comfort and patient engagement; (2) Support during invasive procedures; (3) Humanisation, relationships and the ICU environment; and (4) Perceived contextual influences on healthcare professionals' experience. Participants perceived music therapy primarily in relation to patient comfort, engagement and support during selected procedures and potentially distressing care activities. They also associated its presence with more humanised relationships and a calmer ICU environment. Although the sessions were directed towards patients, some participants described positive contextual influences on their immediate experience while observing or providing care.

**Conclusions:**

Healthcare professionals perceived patient‐directed music therapy as a potentially valuable complementary intervention alongside ongoing ICU care. Within their accounts, its presence was associated with patient comfort and engagement, support during selected procedures and potentially distressing care activities, more humanised and relational care and a calmer care environment. Some participants also described perceived positive contextual influences on their immediate experience while observing or providing care.

**Implications for Nursing Practice:**

These findings provide considerations for nursing practice when patient‐directed music therapy is being considered in ICU care. Nurses may contribute to assessing the patient's clinical condition, willingness and capacity to participate, identifying or communicating musical preferences, coordinating the timing of sessions with the multidisciplinary team and facilitating referral to a qualified music therapist. Careful planning is required to ensure that sessions do not interfere with clinical monitoring, necessary procedures or patient safety.

**Reporting Method:**

The study was reported in accordance with the Consolidated Criteria for Reporting Qualitative Research (COREQ) to enhance transparency and methodological rigour in qualitative studies involving interviews.

**Patient or Public Contribution:**

No patient or public contribution.

## 1 Introduction

Intensive care units are highly technological environments in which critically ill patients may experience discomfort, restricted communication, separation from family members, environmental noise and limited control over their care. Invasive procedures and other potentially distressing care activities may further intensify this experience. Humanising critical care therefore requires attention not only to physiological stability but also to patients' dignity, comfort, preferences, relationships and subjective experience of care (Alberto Sánchez‐Alfaro et al. 2022; Arias‐Rivera 2017; Rojas 2019).

Music therapy has been introduced into critical care as a complementary, person‐centred approach that can be adapted to patients' clinical condition, responsiveness and musical preferences. Its incorporation into ICU care may provide opportunities for comfort, engagement, expression and relational connection within an environment otherwise dominated by technical interventions. Healthcare professionals have previously described live music in the ICU as potentially contributing to patient comfort, reduced environmental tension and more humanised care (Álvarez 2018).

Healthcare professionals occupy a particularly relevant position from which to interpret the integration of music therapy into ICU practice. During these sessions, they may observe patients' responses, continue to provide clinical care, interact with family members and experience how the presence of music relates to the wider care environment. Previous studies have shown that healthcare professionals' direct exposure to live music or music‐based interventions shapes how they perceive their relevance, acceptability and potential contribution to patient‐centred and humanised care (Álvarez 2018; Thorn et al. 2026). Their accounts may therefore provide insight into how patient‐directed music therapy is understood in relation to the patient experience, care interactions, professional–patient–family relationships and the atmosphere of the ICU.

Although an emerging body of research has examined healthcare professionals' perceptions of live music and other music‐based interventions in hospital and intensive care settings, this literature remains limited and has frequently focused on general attitudes, acceptability or nursing perspectives (Álvarez 2018; Thorn et al. 2026). Less is known about how members of the multidisciplinary ICU team experience and interpret music therapy delivered by a qualified music therapist to critically ill patients during routine care, including selected invasive procedures. In particular, further qualitative understanding is needed of how professionals interpret its relevance to the patient experience, care interactions, the ICU environment and their own situated experience while providing care.

Previous publications from the broader research programme have examined the experiences of critically ill patients receiving music therapy and the perspectives of family members present within the ICU context (Saldaña‐Ortiz, Caballero‐Galilea, et al. 2025; Saldaña‐Ortiz, Martínez‐Miguel, et al. 2025; Saldaña‐Ortiz, Recio‐Rivas, et al. 2025). However, these publications did not examine the separately generated accounts of healthcare professionals who observed or provided care during the patient‐directed sessions. The present study addresses this distinct participant perspective.

## 2 Objective

This study aimed to explore how ICU healthcare professionals experienced and interpreted music therapy delivered to critically ill patients during routine care, including their perceptions of the patient experience, care interactions, the ICU environment and contextual influences on their own professional experience.

## 3 Method

### 3.1 Design

A qualitative study informed by hermeneutic phenomenology was conducted to explore how ICU healthcare professionals experienced and interpreted music therapy delivered to critically ill patients during routine care. This approach was selected because it enables examination of lived experience and interpretation of the meanings that participants attribute to a situated clinical experience within their professional context (Neubauer et al. 2019; Teherani et al. 2015).

### 3.2 Setting and Subjects

The study was conducted in the adult ICU of a second‐level hospital in the Community of Madrid, Spain. The unit has 18 beds and provides care for critically ill adult patients requiring complex monitoring and treatment.

Purposive sampling was used to recruit members of the multidisciplinary ICU team who had been present during a music therapy session delivered to a critically ill patient. Participants were eligible if they had directly observed the session or had provided patient care while it was taking place.

Fourteen healthcare professionals participated in the study. The sample included nurses, physicians and nursing assistants with varying levels of ICU experience. Recruitment continued until sufficient depth and repetition in the data indicated that no substantially new insights were emerging.

The patient‐directed intervention consisted of live, individualised music therapy sessions delivered by a qualified music therapist using voice and/or guitar. Each session was adapted to the patient's clinical condition, responsiveness and musical preferences. Whenever possible, patients contributed to the selection of music; when they were unable to communicate their preferences, family members provided this information where available. Music therapy sessions were delivered as part of the approved research protocol alongside ongoing ICU care. Some sessions coincided with clinically indicated procedures or care activities when considered appropriate by the clinical team. The socio‐demographic data collected from the study participants were necessary to understand the context and to allow the transferability of the results to other places and contexts. A total of 14 interviews were conducted with healthcare professionals (*n* = 14): 10 women and 4 men where they were coded as ‘PROF’ to maintain the anonymity.

Recruitment and preliminary analysis proceeded iteratively. By the completion of the 14 interviews, increasing repetition was evident across participants' accounts, and the dataset was considered to provide sufficient depth and variation to address the study aim. This decision was based on the adequacy of the information generated rather than on an assumption that no further interpretations were possible.

This study formed part of a broader doctoral research programme examining patient‐directed music therapy in the ICU from the perspectives of patients, family members and healthcare professionals. The same group of critically ill patients received the music therapy sessions considered across the related studies, and the studies shared the clinical setting and intervention protocol. Two previous publications drew on the same corpus of interviews with 14 patients, examining their experiences of music therapy in relation to invasive procedures and their broader perceptions of illness and recovery, respectively (Saldaña‐Ortiz, Martínez‐Miguel, et al. 2025; Saldaña‐Ortiz, Recio‐Rivas, et al. 2025). A further publication reported findings from a separate corpus of interviews with family members (Saldaña‐Ortiz, Caballero‐Galilea, et al. 2025). The present study reports exclusively on a separately generated corpus of interviews with 14 healthcare professionals who had been present during one patient‐directed music therapy session. The professional participants, interview guide, research question, data source and analysis were specific to the healthcare‐professional perspective. No patient or family interview data were included in the present analysis.

### 3.3 Data Collection

The healthcare‐professional interviews constituted a separate data source from the patient and family interviews reported in the related publications. Each professional participated in one individual semi‐structured interview conducted (Table 1) 24–48 h after the patient‐directed session they had attended. The interview guide was specifically developed to explore professionals' observations and interpretations of the patient experience, care interactions, the ICU environment and their own situated experience while observing or providing care.

**TABLE 1:** Interview guide.

Topics	Questions
Perceptions of the care environment during music therapy	Sample questions: What is your perception of the climate in the Unit after the music therapy? Aspects to explore: To explore healthcare professionals' perceptions of the ICU environment and care atmosphere during and after music therapy sessions delivered to patients.
Healthcare professionals' experience while providing care during music therapy	Sample questions: What do you feel when you listen to music that makes you feel more relaxed? How does music affect your comfort in the ICU? Aspects to explore: To explore how healthcare professionals experienced the presence of music therapy while observing or providing care.
Music therapy within ICU care and humanisation	Sample questions: What is your overall impression of the intensive care unit? How do you perceive the atmosphere on the ward after music therapy? Aspects to explore: To explore how healthcare professionals interpret the role of music therapy within ICU care, relationships, and the wider care environment.

*Note:* Semi‐structured interview guide designed to explore how healthcare professionals experienced and interpreted patient‐directed music therapy in the ICU, including perceptions of the care environment, patient experience and their own situated experience while providing care.

### 3.4 Data Analysis

Data were analysed using Braun and Clarke's reflexive thematic analysis (Braun and Clarke 2006; Braun and Clarke 2019). The analysis was primarily inductive, with codes developed from the healthcare‐professional interviews rather than from a predetermined coding framework. Initial coding was predominantly semantic and focused on participants' explicit accounts, while the hermeneutic phenomenological orientation supported their interpretation within the relational, professional and clinical context of ICU care.

The principal researcher led the analysis. Familiarisation involved repeated reading of the transcripts and the preparation of reflexive notes to examine initial interpretations and the potential influence of the researcher's clinical background and prior assumptions. Initial codes were generated across the full healthcare‐professional interview corpus and were iteratively reviewed, refined and grouped according to shared patterns of meaning. Related codes were used to construct candidate themes, which were recursively examined against the coded extracts, individual transcripts and the dataset as a whole. The themes were subsequently refined, defined and named in relation to the study aim. Movement between these analytic activities was recursive rather than a fixed linear sequence.

The hermeneutic phenomenological orientation informed the analytic movement between specific excerpts, participants' complete accounts and the dataset as a whole. Developing interpretations were discussed within the research team to consider alternative readings and challenge prior assumptions, rather than to establish coding agreement or a single objectively correct interpretation.

Recruitment and preliminary analysis proceeded iteratively. By the completion of the 14 interviews, increasing repetition was evident across participants' accounts, and the dataset was considered to provide sufficient depth and variation to address the study aim. This decision was based on the adequacy of the information generated rather than on an assumption that no further interpretations were possible.

Only the healthcare‐professional interview transcripts were included in the analysis reported in this manuscript. Patient and family transcripts, codes, quotations and previously developed themes were not incorporated into the coding or theme‐development process.

### 3.5 Rigour and Reflexivility

Rigour was supported through coherence between the study aim, hermeneutic phenomenological orientation, sampling strategy, data collection and analytical approach. The researchers engaged repeatedly with the interview transcripts and used reflexive memo writing to examine developing interpretations and the potential influence of prior assumptions. Interpretations were discussed within the research team to challenge assumptions and support reflexive engagement with the data. A description of the study context, participants, intervention and data collection procedures was provided to enable readers to assess the transferability of the findings. Reporting was guided by the Consolidated Criteria for Reporting Qualitative Research (COREQ) (File S1) (Moser and Korstjens 2018; Tong et al. 2007).

The interviews were conducted by the principal researcher, a nurse with professional experience in critical care and training in qualitative research. The researcher had no prior relationship with the participants. Given her clinical background and prior knowledge of music therapy, reflexive memos were used throughout data collection and analysis to examine how her assumptions, professional experience and responses to the data might influence questioning and interpretation. Developing interpretations were discussed within the research team to challenge assumptions and support reflexive engagement with the data.

### 3.6 Ethical Considerations

The broader research project, including the patient‐directed music therapy sessions and the collection of interview data from patients, family members and healthcare professionals, was approved by the Research Ethics Committee of Hospital Universitario Rey Juan Carlos (reference CI 23.238). Music therapy was delivered as part of the approved research protocol and was not part of the ICU's usual service provision.

Before each music therapy session, patients received verbal and written information about the study. Patients who were able to sign provided written informed consent. When a patient was unable to provide a signature, the study was explained to the patient, verbal consent was obtained, and a family member signed the consent form as the patient's representative. A single consent process covered all research activities applicable to the participant.

The invasive procedures or care activities taking place during some music therapy sessions were clinically indicated components of routine ICU care and were not introduced or modified for research purposes. Consent to participate in the music therapy study did not replace the usual clinical decision‐making and authorisation procedures applicable to those care activities.

Healthcare professionals received written and verbal information about the study and provided written informed consent before the patient‐directed music therapy session they attended. Their consent covered participation in the study and the subsequent audio‐recorded interview. Participation was voluntary, and interview recordings and transcripts were handled confidentially and pseudonymised using study codes.

## 4 Results

Fourteen healthcare professionals participated in the study, including nurses, physicians and nursing assistants with varying levels of ICU experience. Each participant had been present during one patient‐directed music therapy session and subsequently completed one individual semi‐structured interview (Table 2). Reflexive thematic analysis generated four themes describing how participants experienced and interpreted patient‐directed music therapy during ICU care: (1) Comfort and patient engagement; (2) Support during invasive procedures; (3) Humanisation, relationships and the ICU environment; and (4) Perceived contextual influences on healthcare professionals' experience. Across the themes, participants primarily interpreted music therapy in relation to the patient's experience and the delivery of care, while also describing how its presence shaped the immediate relational and emotional context in which care was provided. Overall, participants described music therapy primarily as a complementary intervention that benefited patients and improved the atmosphere of care.

**TABLE 2:** Sociodemographic data.

Coding	Age	Gender	Professional role	ICU experience, weeks	Clinical activity or participant role during the session
PROF1	24	Woman	Nurse	76	BIPAP placement
PROF2	31	Woman	Nurse	520	Extubation
PROF3	29	Woman	Nursing Assistant	364	Mouthwash
PROF4	26	Woman	Nurse	4	Central Venous Cure
PROF5	35	Man	Physician	3	Observer^a^
PROF6	26	Woman	Nurse	3	Cleanliness
PROF7	26	Man	Nursing Assistant	32	Cleanliness
PROF8	30	Woman	Nurse	104	Nursing Technician
PROF9	32	Woman	Nurse	76	Nursing Technician
PROF10	40	Woman	Physician	780	Extubation
PROF11	25	Woman	Physician	2	Observer^a^
PROF12	55	Woman	Nurse	External ICU staff	Dialysis
PROF13	54	Man	Physician	1300	Observer^a^
PROF14	24	Man	Nurse	36	Nursing Technician
Mean	32.64			253.85	

*Note:* Descriptive information of the healthcare professionals participating in the study, including age, sex, professional role, length of experience in the ICU (in weeks), and the type of procedure observed or performed during the music therapy sessions.

^a^ Observers were professionals present during the session but not directly engaged in clinical procedures.

### 4.1 Comfort and Patient Engagement

Participants described music therapy as creating moments in which patients appeared more relaxed, animated or emotionally engaged with their surroundings. These interpretations were based on professionals' observations during the sessions rather than on formal assessments of patient anxiety, mood or clinical outcomes. Participants associated patients' responses to the music with a temporary shift away from the monotony and restrictions of the ICU and towards a more personally meaningful experience.

In recovery, apart from drugs and medicine, the state of mind and attitude is very important and I think that music leads to a good attitude and to being more cheerful. The ICU is a very boring place, so I think it's good. (PROF14)

### 4.2 Support During Invasive Procedures

Participants described music therapy as a form of support during selected clinical procedures and potentially distressing care activities. Within their accounts, patients appeared to direct their attention towards the music, which professionals interpreted as helping to make the experience less intrusive and more manageable. Some participants also perceived patients as more relaxed or cooperative while care was being provided.

After the music therapy we asked her and she said that she was delighted, that she loved it. While we were removing the tube, it was as if she didn't know about that part; she was listening to the music, and she didn't know anything about it. She tells it as if she hadn't lived through that part. (PROF1)

It was positive, relaxed, with the patient. I noticed that maybe the patient was happy… it's not the same as a patient who is tense. I think the music also relaxes them, so it makes it easier for you to work with the music. (PROF3)

These accounts suggest that professionals perceived music therapy as supporting distraction, comfort and interaction during procedures. The study did not objectively assess pain, anxiety, physiological responses, procedural success or patient cooperation.

### 4.3 Humanisation, Relationships and the ICU Environment

Participants interpreted music therapy as introducing a more personal and relational dimension into an environment otherwise characterised by technology, clinical routines, noise and restricted social interaction. Music was described as shifting attention towards the patient as a person and creating opportunities for shared experiences among patients, family members and healthcare professionals.

I believe that all of this can improve the patient a lot. Apart from their clinical observations—and I have noticed this less—it is true that, above all, the isolation and loneliness they experience are reduced a little with music therapy. (PROF7)

I found it very amusing, very pleasant, to see the patient so animated. The relatives were also there collaborating as if it was the most important thing at that moment. It seemed to me a very effective way of disconnecting a little from the illness situation. They were happy, they were comfortable, and I think they had a good time. (PROF9)

Within participants' accounts, the presence of music therapy was associated with a less impersonal atmosphere and with moments of connection involving the patient and, when present, family members. These experiences were interpreted by professionals as consistent with a more humanised approach to ICU care.

### 4.4 Perceived Contextual Influences on Healthcare Professionals' Experience

Although the music therapy sessions were directed towards patients, some participants described contextual influences on their own experience while observing or providing care. They reported that the presence of music could make a demanding moment feel calmer, more pleasant or more manageable. These accounts concerned the immediate experience of care and were not interpreted as evidence of changes in occupational stress, burnout, health, productivity or professional performance.

I think that positively, always on days when you have a lot of work or a heavy workload, it makes that little while more pleasant, more bearable. At that moment, it distracts you; you are focused on what you are doing, but at the same time it takes you a little away from the bustle, from the number of things you have to do… I do like it. (PROF1)

Well, I felt relaxed, I felt at ease. María comes with me and puts on some music while we are caring for the patient, and we cheer each other up. It's like you make everything livelier. (PROF3)

Participants' descriptions suggest that the presence of music therapy may have shaped the immediate emotional climate surrounding care. However, these situated perceptions should not be understood as measured effects on professionals' psychological or occupational outcomes.

## 5 Discussion

This study explored how ICU healthcare professionals experienced and interpreted music therapy delivered to critically ill patients during routine care. Participants' accounts were organised into four themes: comfort and patient engagement; support during invasive procedures; humanisation, relationships and the ICU environment; and contextual effects on healthcare professionals' experience. Overall, participants interpreted music therapy primarily in relation to the patient's experience and the delivery of care. They also described how its presence shaped the immediate relational and emotional context in which care took place.

Participants perceived patients as more relaxed, animated or engaged during the music therapy sessions. These observations are consistent with previous research showing that ICU professionals may view live music as contributing to patient and family comfort and as having a potential place within humanisation initiatives (Álvarez 2018). More recent evidence concerning patient‐tailored live music similarly indicates that nurses perceive such interventions as creating meaningful moments for patients (Thorn et al. 2026). However, the present findings represent professionals' interpretations of patients' responses. Patient anxiety, comfort, mood and engagement were not formally assessed, and the findings should not be interpreted as evidence of clinical effectiveness.

Music therapy was also perceived as providing support during selected procedures and potentially distressing care activities. Participants described patients directing their attention towards the music and appearing more relaxed or cooperative while care was being provided. This finding is broadly consistent with research in other clinical populations suggesting that music therapy may be perceived as a useful source of distraction and support during painful or invasive procedures (Giordano et al. 2020). Nevertheless, differences in population and setting limit direct comparison: Giordano et al. studied paediatric oncology patients, whereas the present study examined professionals' accounts of adult ICU care. Furthermore, no objective measures of pain, anxiety, physiological response, procedural tolerance or cooperation were obtained. The findings therefore indicate perceived procedural support rather than demonstrated symptom reduction.

The findings also suggest that professionals interpreted music therapy as introducing a more personal and relational dimension into the ICU. Participants associated the sessions with moments of shared attention, family involvement, reduced impersonality and recognition of the patient beyond their clinical condition. This interpretation is consistent with ICU humanisation literature that emphasises dignity, relational care, patient and family involvement and attention to the experience of critical illness (Alberto Sánchez‐Alfaro et al. 2022; de la Fuente‐Martos et al. 2018; Padilla‐Fortunatti et al. 2018). In the present study, however, patients and family members were not interviewed. References to their comfort, involvement or emotional responses must therefore be understood as interpretations offered by healthcare professionals.

Although the sessions were directed towards patients, some participants described contextual influences on their own experience while observing or providing care. They reported that music could make a demanding moment feel calmer, more pleasant or more manageable. Previous studies have similarly found that healthcare professionals' direct exposure to music‐based interventions can shape their perceptions of the care environment and of the relevance of music within clinical practice (Álvarez 2018; Thorn et al. 2026). These findings should be interpreted as situated accounts of the immediate care context. The present study did not evaluate occupational stress, burnout, compassion fatigue, health, productivity, job satisfaction or professional performance and cannot establish an effect on these outcomes.

Taken together, the findings indicate that healthcare professionals perceived patient‐directed music therapy as potentially compatible with person‐centred and humanised ICU care. Its perceived value was related not only to the music itself but also to its adaptation to the patient, the clinical situation and the interactions occurring during the session. Integration into practice would therefore require attention to patient preferences, clinical appropriateness, communication with the multidisciplinary team and the role of the qualified music therapist. These findings support consideration of music therapy as a complementary component of care rather than as a substitute for established clinical or pharmacological interventions.

### 5.1 Strengths and Limitations

A strength of this study was its focus on the situated experiences of members of the multidisciplinary ICU team who had been present during patient‐directed music therapy. The hermeneutic phenomenological orientation enabled attention to the meanings participants attributed to the sessions within their clinical context. However, the study was conducted in a single ICU with a small purposively selected sample, which may limit the transferability of the findings. Each participant reflected on one session, and the clinical circumstances, care activities and level of involvement varied across participants.

Some questions in the semi‐structured interview guide referred explicitly to relaxation, comfort or changes in the ICU atmosphere and therefore presupposed potentially positive effects of music. These formulations may have encouraged participants to focus on positive experiences or may have shaped how they described their own comfort and relaxation. Findings concerning contextual influences on healthcare professionals' experience should therefore be interpreted in relation to the wording of the interview questions and as situated participant accounts rather than as evidence of an effect on professional well‐being.

In addition, accounts of patient and family responses were provided by healthcare professionals rather than directly by patients or family members within this study. The study did not include objective clinical or occupational measures and cannot establish the effectiveness of music therapy or its longer‐term influence on patients, families, professionals or ICU practice.

### 5.2 Recommendations for Future Research

Future research should examine the long‐term and repeated use of music therapy in ICUs and include multicentre studies to enhance transferability. Mixed‐methods and longitudinal designs are recommended to assess both experiential and clinical outcomes, as well as tailored interventions based on patient and contextual characteristics.

### 5.3 Implications for Nursing Practice

The findings provide insight into how healthcare professionals experienced and interpreted patient‐directed music therapy within ICU care and may inform consideration of how such interventions could be carefully integrated and further evaluated. They do not establish clinical effectiveness or support routine or widespread implementation. Any consideration of music therapy in the ICU should remain responsive to the patient's clinical condition, preferences, willingness and capacity to participate.

Nurses may contribute to identifying relevant patient preferences, assessing the patient's immediate clinical condition and ability to engage, communicating with family members when appropriate and coordinating the timing of sessions with the multidisciplinary team. Where music therapy is considered clinically appropriate, referral should be made to a qualified music therapist, who is responsible for assessing, designing and delivering the intervention. Interprofessional planning is required to ensure that sessions are coordinated with ongoing care and do not compromise clinical monitoring, necessary procedures, communication among staff or patient safety.

The professionals' accounts suggest that the timing of music therapy in relation to selected procedures and care activities warrants further evaluation. Future implementation studies should examine feasibility, acceptability, safety and patient‐reported and clinical outcomes before wider adoption within ICU practice.

## 6 Conclusions

Healthcare professionals perceived patient‐directed music therapy as a potentially valuable complementary component of ICU care. Within participants' accounts, its presence was associated with patient comfort and engagement, support during selected procedures and potentially distressing care activities, and a more humanised, relational and welcoming ICU environment. Some participants also described perceived positive contextual influences on their immediate experience while observing or providing care.

These findings represent healthcare professionals' situated experiences and interpretations rather than evidence of clinical or occupational effectiveness. Patient and family responses were reported indirectly through professionals' accounts, and the study did not assess patient anxiety, pain, physiological outcomes, professional stress, burnout or well‐being. The findings may inform consideration of how patient‐directed music therapy could be carefully integrated and further evaluated when clinically appropriate, consistent with the patient's preferences and willingness to participate, delivered by a qualified music therapist, and coordinated with the multidisciplinary team. Further evaluation is required before wider implementation in ICU practice.

## Author Contributions

**Verónica Saldaña‐Ortiz:** conceptualization, investigation, methodology, data curation, writing – review and editing, writing – original draft, formal analysis. **María Caballero‐Galilea:** writing – original draft, writing – review and editing. **Vanesa Intriago‐García Consuegra:** writing – original draft, writing – review and editing. **José Miguel Mansilla‐Domínguez:** conceptualization, investigation, writing – review and editing, supervision, methodology, formal analysis. **Beatriz García‐Fernández:** writing – original draft, writing – review and editing. **Esther Martínez‐Miguel:** validation, visualization, formal analysis.

## Funding

The authors have nothing to report.

## Ethics Statement

The study had the approval of the Ethics Committee (CI 23.238).

## Consent

The authors have obtained informed consent from the patients and/or subjects referred to in the article. This document is held by the corresponding author.

## Conflicts of Interest

The authors declare no conflicts of interest.

## Supporting information


**File S1:** Consolidated Criteria for Reporting Qualitative Research (COREQ). Modified from Tong et al. (2007).

## Data Availability

The interview data that support the findings of this study are available from the corresponding author upon reasonable request. The data are not publicly available because they contain information that could compromise participant privacy and are subject to the conditions established by the ethics approval and informed‐consent process.
