# Supplementary material for: The Integration of Music Therapy in the Intensive Care Unit: Health Care Professionals' Experience of an Emerging Paradigm
Source: Nurs Open. 2026 Nov 3;13(11):e70994. doi: 10.1002/nop2.70994 (PMC13731725; doi:10.1002/nop2.70994)
Supplement: Supplementary file 1 [file NOP2-13-e70994-s001.docx]

**Supplementary File 1. Consolidated Criteria for Reporting Qualitative Research (COREQ)**. Modified from Tong et al (2007).

| **Theme** | **Guiding question/ description** | **Reported on page** |
| --- | --- | --- |
| **Domain 1. Reflexivity and the research team** | | |
| ***Characteristics of interviewing staff*** | | |
| 1.Interviewer/facilitator | Which author(s) conducted the interviews? | VSO (Page 8) |
| 2. Credentials | What were the Researcher's credentials? | Master in Critical Care |
| 3. Occupation | What was your occupation during the study? | Emergency Nurse |
| 4. Gender | Is the researcher a man or a woman? | Woman |
| 5. Experience and training | What experience does the Researcher have? | 14 years as a Nurse in different health care settings |
| ***Relationship with participants*** | | |
| 6. Established relationships | Was it an established relationship before the study? | No. Participants who were currently in the Unit were selected. |
| 7. Participant's Knowledge of the Research | What did the participants do that the researcher knows about, e.g. personnel, objectives, reasons for doing the research? | Before consent, participants were informed that the interviewer was the principal researcher and a nurse conducting the study as part of a doctoral research programme. They received information about the study purpose, the voluntary nature of participation, the interview procedure, confidentiality, audio-recording, and their right to withdraw. |
| 8. Researcher Characteristics | What characteristics were reported about the interviewer? For example, alleged motives and interests in the research topic? | The principal researcher was a nurse with experience in critical care and qualitative research training. She had a prior academic and clinical interest in music therapy and humanised ICU care. Reflexive notes were used to examine how her professional background and assumptions might influence data collection and interpretation. |
| **Domain 2. Study design.** | | |
| ***Theoretical Framework*** | | |
| 9. Theory and Methodological Orientation | What methodological orientation underpins the study? For example, grounded theory, discourse analysis, ethnography, phenomenology, content analysis, etc. | Hermeneutic phenomenology |
| ***Selection of Participants*** | | |
| 10. Sampling | How were participants selected? For example, purpose-based, convenience, consecutive, snowball. | Purposive sampling was used to recruit members of the multidisciplinary ICU team who had been present during one patient-directed music therapy session. |
| 11. Selection method | How were participants contacted? For example, face-to-face, telephone, email. | Participants were contacted face-to-face to explain what the research was about. |
| 12. Sample Size | How many participants were in the study? | 14 (Page 5 and page 9) |
| 13. Abandonments | How many people dropped out of the study and what reasons did they give? | No invited healthcare professionals declined participation or withdrew after enrolment. |
| ***Adjustments*** | | |
| 14. Adjustments to data collection | Where were the data collected? For example, at home, at the clinic, at your workplace. | Fourteen healthcare professionals participated: 10 women and 4 men, including nurses, physicians, and nursing assistants. The mean age was 32.64 years, with a range of 24–55 years. Participants had varying levels of ICU experience. |
| 15. Presence of non-participants | Was anyone else present besides the participants and the researcher? | No one else was present. The researcher and the participants at the time of the interviews were alone. |
| 16. Description of the sample | What are the main characteristics of the sample? | Of the participants (n=14):   - HEALTHCARE PROFESSIONALS: (n=14) 10 women and 4 men. The age measure was 24 years. |
| ***Data collection*** | | |
| 17. Interview guide | Were the questions, instructions, guidelines created by the authors? Was it pilot-tested? | A semi-structured interview guide was developed by the research team in relation to the study aim and relevant literature. The guide is reproduced in Table 1. Some questions explicitly referred to relaxation or comfort; their potentially leading formulation is acknowledged in the limitations. (pages 6 and 7) |
| 18. Repetition of interviews | Were repeated interviews conducted? If so, how many? | No interviews were repeated. |
| 19. Audio/visual recording | Was an audio or visual recorder used to collect the data? | All interviews were audio-recorded with participants’ written consent and transcribed verbatim. Photographs or videos of the music therapy sessions were not included in the dataset analysed for this manuscript. |
| 20. Field notes | Were field notes made  during/after the visit? | Field notes were taken during the interview. Similarly, the researcher's notes were subsequently taken on aspects such as method, theoretical content and the course of data collection. |
| 21. Duration | What was the duration of the interviews? | An average of 40 minutes per interview. |
| 22. Data saturation | Was data saturation discussed? | Recruitment and preliminary analysis proceeded iteratively. Increasing repetition was evident across the later interviews, and the dataset was considered sufficiently rich and varied to address the study aim. The study does not claim that no further interpretations were possible. |
|  |  |  |
| 23. Return of transcripts | Were the transcripts returned to the participants for comments and/or corrections? | No |
| **Domain 3: Analysis and recommendations** | | |
| ***Data analysis*** | | |
| 24. Number of data encoders | How many data coders coded the data? | One researcher led the initial coding. (Pages 7 and 8) |
| 25. Description of coding | Do the authors provide a description of the coding tree? | Codes and themes were subsequently discussed within the research team to examine alternative interpretations rather than to establish coding agreement. |
| 26. Topic Referral | Are issues decided in advance or derived from the data? | Coding was primarily inductive and predominantly semantic. |
| 27. Software | What software, if any, did you use to manage the data? | ATLAS.ti 22 was used to support data organisation and management. |
| 28.Checking of participants | Do participants provide feedback on the findings? | NO |
| ***Reports*** | | |
| 29. Submission of quotations | Are participants quoted to illustrate the issues/results? | Participants are cited, with alpha-numeric codes in each of the citations provided in the report of results. |
|  | Was each quote identified, e.g. participant number? | Each appointment was identified with its participant number. Provided in the report of results |
| 30. Consistency between data and results | Is there consistency between the data presented and discussion? | Yes. See results and Discussion |
| 31. Clarity of the main issues | Were the main issues clearly presented in the Outcomes? | Yes, see results (Pages 9, 10, 11 and 12) |
| 32. Clarity of Minor Issues | Is there a description and discussion of minor issues? | Not applicable. The analysis generated four main themes, and no formal subthemes were reported. |
